# Mr. Whately on Luxations of the Humerus

**Published:** 1805-06-01

**Authors:** Tho. Whateley

**Affiliations:** Grafton-street


### *To the Editors of the Medical and Physical Journal.*

Gentlemen,

I HAVE lately succeeded in reducing two luxations of the humerus (the head of the bone being in the axilla) by the following simple method; and though it does not differ in its principle, nor much in the method, from some

2 others others usually recommended, it may perhaps be found useful, in some recent cases of this accident.

Let the patient be seated on a chair of the usual height; then take a common soft towel, or table-cloth, put it round his body, just beneath the axilla of the dislocated limb; let each end be held by a stout assistant, seated on a chair close to the patient; tie another soft towel upon the dislocated arm, in a very firm manner, just above the elbow, and let both ends of it, when tied, be of a length sufficient to be held firmly by two other men, sitting on chairs directly opposed to the former: let the fore-arm be then bent, and kept in this position by a fifth assistant, standing; the arm being placed at a right angle from the body. Let the surgeon now seat himself on a chair, or stool, about six inches higher than that on which the patient is seated; let him sit on the outside of the patient's arm, opposite to the dislocated shoulder, with his right foot placed on the patient's chair, so as to raise his knee nearly into the axilla; let him then take a ball of fine packthread, wound together in a solid manner, of the size of a man's fist, and place it (previously wrapped in linen rag) in the patient's axilla, directly under the head of the dislocated humerus, keeping it in this situation with his knee. Let the two assistants, holding the towel tied round the arm, be next directed to make a steady, progressive, and strong extension of it; and let those placed on the opposite side keep the patient's body firmly seated on the chair in its natural position, neither pulling so strongly as to incline it towards them, nor with so little force as to suffer their antagonists to draw it to the other side. When this extension, and counter-extension, have been thus gradually made, with all the force the assistants can use, for a few seconds, so as to convince the surgeon that the head of the dislocated humerus is probably brought on a level with the edge of the glenoid cavity of the scapula, let him, at this instant, raise the ball in the axilla with his knee, by a strong muscular exertion, which the position of his foot on the chair will enable him to do in a very forcible manner; at the same time, let the palm of one hand be pressed firmly on the acromium, so as to prevent the body from being raised upwards, whilst the humerus, firmly grasped near the elbow with the other, is depressed. If these united exertions be made steadily, and at the same instant, I am confident that an intelligent surgeon will, for the most part, succeed in reducing a recent luxation of the humerus. I have already stated, that I have lately succeeded in two cases very readily; the first had been been dislocated a few hours only, and the last about a week.

Two or three years previous to my having practised this method, I had reduced three dislocated shoulders, by suspending the body very gently and gradually by double pullies; one of which cases had been dislocated near a month. In the first case, the head of the bone slipped into its place the moment the patient was suspended: in the second, while in this position, he became faint, but the reduction was not effected by the suspension; at the instant, however, that his toe touched the ground, on being let down, and before he had recovered from his swoon, I took advantage of this situation, and succeeded in reducing the shoulder, by putting the fist of one hand into the axilla, and pushing the head of the humerus upwards with a little jerk, while, with the other, I depressed this bone, at the same instant, by taking hold of its lower part, near the elbow. In the third case, the suspension by the pulley did not reduce the bone; but, from the success I had had in the preceding case, I put a ball of packthread into the axilla with one hand, and depressing the humerus at the elbow with the other, I succeeded in reducing the limb in the manner already described; this too was done at the instant the patient's toes touched the ground after the suspension.

I am, &c.

*Grafton-street, May 1, 1805.* THO. WHATELEY.

